# “I Feel Like a Fraud Who Acts Like a Feminist”: The Discussion Themes and Sexual Scripts in the Porn Free Women Online Forum

**DOI:** 10.1007/s10508-024-02858-w

**Published:** 2024-04-18

**Authors:** Xinyu Zhang, David E. Silva

**Affiliations:** School of Communication Studies, Kent State University, 300 Midway Drive, Office 201J, Kent, OH 44242 USA

**Keywords:** Sexual script theory, Sexual health, Online health communities, Female sexuality, Pornography, Critical discourse analysis

## Abstract

Research on online pornography abstinence movements has predominantly focused on men’s perspectives, often within the context of the broader manosphere. This focus has overshadowed the unique experiences and viewpoints of women in these movements. Our study aimed to fill this gap by exploring women-centric perspectives in pornography abstinence forums, particularly Porn Free Women (r/pornfreewomen). Using a mixed methods approach, this study examined the sexual scripts presented in women-dominated pornography abstinence communities. Our structural topic modeling analysis delineated the interplay of therapeutic, heteronormative, and empowerment themes that were evident in women’s narratives and expressions. Further, our discourse analysis elucidated three specific scripts: the addiction script, the heterosexual script, and the liberation script. These interweaving narratives show that discussions of women’s pornography abstinence are multifaceted and include a variety of perspectives to negotiate. These results contribute to a nuanced understanding of the values of health and well-being, sexual liberation, and feminism within women’s pornography abstinence communities.

## Introduction

Although *NoFap* is the most popular Reddit forum on the topic of “porn addiction and compulsive sexual behavior recovery” (r/NoFap, 2023), anti-pornography discussions on Reddit predate the NoFap community. The *pornfree* subreddit (320,000 members; r/pornfree, 2023) was created in 2008, just 3 years after Reddit was founded and 3 years before the NoFap forum was created. Pornfree and NoFap share a common anti-pornography stance (Burnett, 2022), but members of pornfree focus on overcoming pornography addiction and counteracting the harms of excessive pornography exposure, particularly for young men. Stopping pornography use is claimed to “reboot” a man’s brain’s reward system to cure pornography-induced dysfunction, an idea paralleled in the broader NoFap movement.

This focus on male biology aligns with estimated forum member demographics, but presents issues for women who wish to engage in discussions on anti-pornography or seek support for their own pornography avoidance. Reddit’s NoFap forum has over one million members (r/NoFap, 2023), but it is estimated that only 5% of users identify as women (Bishop, 2019). Bishop reports the discomforting experience of a woman participating in online communities dominated by men and their experience with pornography and masturbation. The gendered focus and the gender imbalance in the dialog hosted on pornfree prompted some women to establish a space dedicated to their own perspectives. The Porn Free Women forum (13,000 members; r/pornfreewomen, 2023) is a women-only community that offers a safe space for discussing the challenges of abstaining from pornography while catering to the unique experiences and perspectives of female participants.

Research seeking to understand pornography abstinence has predominantly focused on traditional masculine perspectives (e.g., Fernandez et al., 2021; Hartmann, 2021). Less attention has been paid to how women negotiate these issues in the context of movements largely centered on men’s experiences. When discussed, the prevalence of sex-positive feminism, masturbation, and pornography consumption are often regarded as supporting political and sexual liberation by empowering women to challenge dominant discourses and institutions that regulate women’s sexuality. Consequently, women’s perspectives on pornography and masturbation have become part of both “traditionally gendered discourse” and “a postfeminist discourse” (Binswanger & Davis, 2019). However, this framing inadvertently sidelines women who advocate for abstinence from pornography.

This study explores how Porn Free Women members negotiate the tensions inherent in their position. These women are sidelined by online anti-pornography movements dominated by men and they diverge from the discourse offered by sex-positive feminism. In our research, we shed light on these women’s perspectives of pornography abstinence and contribute to a more nuanced discourse around the intersections of addiction, heteronormativity, and liberation. To this end, we focus on the sexual scripts present in the discussions hosted on Porn Free Women. We present how these scripts are employed in discussions on pornography abstinence and how these scripts operate to navigate a complex and ambiguous perception of social and individual expectations. Specifically, we propose that the sexual scripts present on Porn Free Women offer ways to maintain, resist, and transform social norms while also contributing to the internal turmoil expressed by the women in this community.

We begin by conducting a review of research focused on the online anti-pornography movement, which has been predominantly focused on heterosexual, masculine voices. This is followed by a review of the literature of diverging feminist narratives on pornography consumption and the unique position of women within the online anti-pornography movement. We then apply structural topic modeling to comprehensively understanding of how women discuss their experience and perspectives on pornography abstinence on the Porn Free Women forum. Additionally, critical discourse analysis of the texts of these discussions is employed to investigate how sexual scripts intersect with the digital *manosphere*, which is known for its hostile attitudes toward women and promotion of misogynistic ideologies (Hartmann, 2021). By amplifying these women’s voices, we contribute to the body of literature pertaining to women’s perceptions and insights regarding their involvement in the anti-pornography movement.

### Masculine Hegemony in Pornography Abstinence Forums

Pornography abstinence forums aim to alleviate perceived deleterious effects caused by solitary sex by refraining from pornography and masturbation. The prevalence of easily accessible online pornography has led many young individuals to report problematic pornography use, sparking a self-intervention movement to abstain completely from pornography use, and, for *no-fappers*—members of the NoFap community—abstention from masturbation (Chasioti & Binnie, 2021). These movements have attracted a large number of men during recent years and subsequent research attention focused on NoFap as a health community. For instance, Osadchiy et al. (2020) found that men who expressed anxiety in their NoFap posts were concerned about erectile dysfunction and lower libido caused by pornography consumption.

Scholarly work has also delved into the impact of religiousness on perspectives toward anti-pornography and reboot communities. Grubbs et al. (2020) found religiousness can act as a moderator in the relationship between pornography consumption and self-reported pornography addiction, suggesting that higher levels of religiousness amplify the correlation between pornography use and self-reports of addiction. Conversely, Fernandez et al. (2021) concluded that moral incongruence ranked among the least common reasons for abstaining from masturbation. Similarly, Chasioti and Binnie (2021) proposed that members’ perceptions were predominantly shaped by “gender performativity, ongoing self-improvement, and successful social exhibitionism,” overshadowing the influence of religiousness. This juxtaposition of findings underscores the multifaceted motivations within reboot communities, intertwining moral, neurological, and sociocultural factors in a complex web of behavioral determinants.

A handful of research articles have looked at the discussion themes prevalent in pornography abstinence communities. Existing research finds that these predominantly male-dominated communities are permeated with themes of toxic masculinity and misogynistic discourse. Taylor and Jackson (2018) conducted a discourse analysis of these communities and revealed anti-feminist themes in discussions instigated by heterosexual men. Their study suggests men justified their anti-pornography and anti-masturbation stance by deploying evolutionary discourses and constructs of innate masculinity.

Through examining the lived experiences of individuals who report problematic use with pornography, Chasioti and Binnie (2021) demonstrated how narratives concerning adverse childhood experiences, unsatisfactory gender performativity, and unfulfilled lifestyle aspirations coalesce to shape a sense of “being weak,” subsequently inciting feelings of distress. They further pointed out these personal narratives of distress are intimately entwined with a highly rigid culture wherein practices of abstinence are constructed as a means of asserting masculine honor (Chasioti & Binnie, 2021). Furthermore, Smith et al. (2022) conducted an analysis of posts within NoFap communities throughout the COVID-19 pandemic. Their findings suggested that men who participate in these communities often internalize a pervasive feeling of failure and inferiority, measured against what they perceive as a tangible benchmark of their masculinity by attempting the NoFap challenge, typified by prolonged abstinence of masturbation.

These discussion themes have positioned the NoFap community as “an entry point” to a larger digital “manosphere” (Hartmann, 2021, p. 17). The manosphere, a conglomerate of online communities including Men’s Rights Activism (MRA), pickup artists (PUAs), Men Going Their Own Way (MGTOW), Involuntary Celibates (Incels), and The Red Pill (TRP), has been widely documented as particularly misogynistic (Cannito et al., 2021). By analyzing a large dataset of online manosphere content over the past 14 years, Ribeiro et al. (2021) charted the evolution of the manosphere. They found that the newer emerging communities, such as Incels, MGTOW, and TRP, harbor a culture that is infused with toxic misogyny. More interestingly, these communities have the capacity to draw in users from the older established communities such as the PUA and MRA, indicating a flow of ideologies and users within the manosphere. A notable platform that has fostered this growth of the manosphere is Reddit. Massanari (2017) investigated how Reddit’s design, algorithmic configuration, and platform governance indirectly fuel the platform’s role as a “toxic technoculture” purporting anti-feminism and misogyny.

By connecting the NoFap forum to the manosphere, the online anti-pornography discussion has, to a large degree, become about negotiating masculinity. Hartmann (2021) examines the underlying meritocratic heterosexuality within the NoFap movement and highlights the appearance of “manospherian modes of self-relation” within NoFap YouTube videos. In an attempt to better understand the multifaceted masculine identities burgeoning in these digital spaces, Burnett (2022) argued that the NoFap movement’s discursive construction on Twitter represents a political contestation of topics, encompassing nationalism, racism, ableism, and misogyny. Collectively, these studies have critically outlined the masculine subjectivities within the NoFap movement, but they have also led to an understanding of anti-pornography motivations that is heavily limited to the experiences of men.

Increasingly, cyberspace communities have been criticized for facilitating new ways to subordinate women. It has been suggested that the online landscape can perpetuate forms of female subordination through mechanisms of control, such as website filtering and regulation (Yanisky-Ravid & Mittelman, 2016). This hostile environment has been identified as a factor that silences women, thereby inhibiting their active participation in public discussions and debates (Chadha et al., 2020). Notably, online communities dedicated to anti-masturbation and anti-pornography have been particularly associated with environments hostile to women, often endorsing radical ideologies, offensive sentiments, and espousing misogynistic attitudes (Hartmann, 2021).

These online trends run counter to prior anti-pornography movements which have been led by women. Although the roots of modern anti-pornography movements can be traced back to anti-pornography feminists in the 1970s, the voices of women have been sidelined in the largest online anti-pornography communities connected to the manosphere. In a non-systematic search of the last 10 years of posts made to NoFap and pornfree, we found only a handful of references to Mickelwait and Dines and no mentions of Murphy, each of whom is a major contemporary voice discussing anti-pornography. This reversal requires women in online anti-pornography discussions to negotiate perceived medical and sexual health concerns that originate from and are perpetuated by a manosphere that consistently is hostile to women. In the anti-pornography forums dedicated for women, members must address these tensions, articulate their experiences, and interpret their role in the anti-pornography movement. We believe these women are making unique contributions to the discourse on anti-pornography that deserves broader recognition. However, these contributions should be understood in the context of the broader social discussion of anti-pornography.

### Anti-Pornography Movements in Feminist Thought

In the late 1970s and 1980s, a cadre of feminist writers and activists launched an anti-pornography campaign by arguing that pornography perpetuated and reproduced gender-based violence (Bronstein, 2011). It was argued that pornography, with a focus solely on sexually explicit content, contributed to the objectification and submission of women (MacKinnon, 1987). In the USA, the Women Against Pornography (WAP) launched a campaign aimed at raising public awareness of pornography’s and the male-centric sex industry’s detrimental impact. WAP initially gained support from women across the ideological spectrum and had a significant impact on mainstream discourse about pornography (Echols, 2016). Despite its initial success, WAP and the anti-pornography movement gradually disintegrated due to increasing public debate and internal conflicts within the movement (Bronstein, 2011).

“Pro-sex” or anti-censorship feminists like Willis, Rubin, and Vance argued against censoring pornography, saying this amounted to censoring sexual expression and women’s autonomy. Pro-sex feminists posited that an overemphasis on pornography’s danger distracted women from embracing sexual pleasure and produced “carceral” implications by conflating the potential harms of pornography with crimes requiring punishment (Bracewell, 2016). Some other researchers tried to take a scientific view, studying effects of pornography exposure in the lab (Donnerstein et al., 1987). But their research was criticized by both anti- and pro-pornography feminists due to negligence of sociocultural and historical factors in decontextualized experimental research (Ciclitira, 2004).

The Barnard Conference in 1982 brought together a range of feminists to debate pornography. It illuminated the divisions but did not resolve them (Corbman, 2015). In its aftermath, the conference sparked debates on the pleasure and danger associated with pornography. Paasonen (2007) pointed out both anti- and pro-pornography perspectives can limit affective change. Pornography can evoke a spectrum of feelings that go beyond mere disgust or pleasure. As such, Paasonen advocates for a more balanced feminist perspective that acknowledges the multifaceted emotional power of porn without oversimplifying it. This complexity is evident when examining women’s interactions with pornography.

Parvez (2006) conducted interviews with working-class and minority women, revealing their mixed feelings toward pornography. While many women appreciated pornography for its entertainment, arousal, and educational value, they also occasionally felt conflicted, uncomfortable, or even distressed. Similarly, Nikunen (2007) concluded the discourses surrounding pornography, further underscoring its complex nature. It is a topic that elicits varied reactions, with some viewing it as a source of enjoyment and education, while others see it as a potential cause of discomfort and unrealistic sexual expectations (Nikunen, 2007). Pornography is often viewed with ambiguity—it is entertaining yet discomforting, educational yet pressuring. This duality makes pornography a battleground for negotiating tensions, such as the balance between women’s enjoyment of pornography and its potential negative impact (Gurevich et al., 2017).

There has been a growing public acceptance of pornography as a means of exploring sexual gratification and sexual pleasure. Some women report pornographic scenes are effective in enhancing solo and partnered pleasure by provoking physical arousal through voyeurism (Ashton et al., 2019). Similarly, research by Daskalopoulou and Zanette (2020) indicates that some women use feminist pornography to “reach a state of pleasure (exercise of fantasy, sexual gratification) and perfection (optimal way of performing sex)” (p. 982). This points to a growing trend where feminist pornography has emerged as a vehicle to empower women. An analytical study by Fritz and Paul (2017) offers further insight by conducting a content analysis of mainstream and feminist pornography, suggesting that queer feminist pornography contain significantly more signs of female sexual agency. Shor (2022) offers a different perspective, reporting that women are not only likely to actively seek aggression in mainstream pornography but also demonstrate interest in fantasies about practices such as bondage, discipline, dominance, submission, and sadomasochism (BDSM). The women in Shor’s study reported being more aroused by aggressive acts, particularly “hard” aggression, than the male participants. As such, these findings pose a significant challenge to the anti-pornography feminist discourse that predominantly views pornography as problematic.

Negative sentiments toward pornography arise from multiple factors. These include its potential harmful effects on sexual expectations, its often demeaning portrayal of women, and its unrealistic depictions of appearances and practices (Gurevich et al., 2017). Furthermore, Ciclitira (2004) conducted interviews that revealed the influence of the feminist anti-pornography’s stance on women’s views. Ciclitira pointed out that retrospective accounts from the 1970s and 1980s indicate a lasting emotional impact on women, who feel torn between their feminist beliefs and their enjoyment of pornography. Also, the fabrication of “pornography addiction” has intensified concerns. This perceived “excessive and uncontrollable” consumption is increasingly viewed as a public health issue, adding to the anxiety surrounding pornography (Taylor, 2019).

In a nutshell, pro-sex narratives coexist with some feminists’ opposition to pornography. In the meantime, the media and political narratives, employing biomedical scientific terminology, assert that pornography directly harms physical and mental health, further complicating women’s subjective feeling toward it (Burke & MillerMacPhee, 2021). A systematic review conducted by Ashton et al. (2018) collated a myriad of qualitative studies, also documenting the complex and occasionally incongruous self-perceptions of women who consume pornography.

Anti-pornography is characterized by debate, lack of external authoritative conclusions, and competing interpretations, which requires the members of Porn Free Women to engage in a process of sense-making while discussing, negotiating with, and supporting other community members. It is through these communicative behaviors that we seek to understand how the members of Porn Free Women create narratives that help others interpret anti-pornography thought and ultimately make decisions on their sexual, emotional, and relational experiences. To understand the role of these narratives, we apply sexual script theory. This theoretical framework underscores the value of a scripting perspective in understanding how culture informs behaviors by simplifying evolving historical and cultural milieu through the construction of scripts (Simon & Gagnon, 2003).

### Sexual Script Theory

Sexual script theory (SST) posits that sexuality is socially constructed and that social context plays a significant role in shaping romantic relationships and sexual interactions (Gagnon & Parker, 1995). Grounded in social constructionism, SST argues that the metaphorical scripts that guide individuals’ sexual behavior are shaped by social principles (Wiederman, 2015). SST rejects most biologically grounded explanations of sexual behavior and instead refers to socially constructed norms that define the bounds of permissible and expected sexual behaviors and interactions (Simon & Gagnon, 2003). These sexual scripts operate at three hierarchical levels that inform how sexual scripts are understood and performed: cultural scenarios, interpersonal scripts, and intrapsychic scripts (Gagnon & Parker, 1995). Cultural scenarios reflect the societal norms and values pertaining to sexuality and may also influence both the interpersonal and the intrapsychic scripts. Interpersonal scripts allow for the interpretation of cultural scenarios in specific situations. Intrapsychic scripts refer to the individual construction of sexual motivation, sexual desire, and sexual arousal (Gagnon & Parker, 1995). At each level, scripts are both negotiated and selectively performed.

Recent scholarship has explored shifts in the traditional heterosexual sexual scripts (Klein et al., 2019). While cultural norms and gender stereotypes may influence individuals’ perceptions of sexuality, their actual sexual experiences and relationships may deviate from these expectations (McCabe et al., 2010). More egalitarian sexual scripts have emerged, challenging traditional gender roles and submissive scripts for women (Klein et al., 2019). For example, Spišák (2017) identifies four sexual scripts in adolescents’ narratives on pornography use: harm, gendered double standards, the good girl, and pleasure technology. Spišák notes that girls portrayed themselves as having “agency, competence, and volition” when consuming pornography (2017, p. 359). Similarly, Healy-Cullen et al. (2023) leverages feminist perspectives to argue that young women use available sexual scripts to frame themselves as empowered subjects, distinguishing pornography from real-world intimacies. This suggests that women who consume pornography challenge traditional sexual scripts by exhibiting agency and initiative. Additional empirical evidence presented by Braithwaite et al. (2015) suggests pornography consumption does associate with more permissive intrapsychic sexual scripts and these attitudes mediate the relationship between pornography consumption and some sexual behaviors. However, Todesco and Camoletto (2021) argue sexual scripts acquired through women’s pornography consumption must also be activated before being applied to sexual behaviors.

This shift echoes Simon and Gagnon’s (2003) work which moves away from a social learning position toward a social constructionist position. They emphasize the role of individuals as active participants in interpreting and using scripts. Our study builds on this perspective, viewing sexual scripts as dynamic constructs that women continuously reshape. The sexual script theoretical framework gives us the language to discuss what the Porn Free Women members contribute to the community when they take the time to explain their interactions with pornography and their interpretations of their sexual practices. These women, collectively, construct scripts that are shared with others on the Porn Free Women forum and, given the public nature of the forum, other Reddit users. Those reading the discussion on Porn Free Women choose how to interpret, use, and reshape the scripts presented by the collective.

### The Present Study

Collectively, the literature reviewed shows a shift in challenging traditional sexual scripts when women consume pornography. However, due to the male-dominated and often misogynistic online discourse, a gap in our knowledge persists regarding women’s perspectives on abstaining from pornography and the sexual scripts associated with this decision. Women in online anti-pornography spaces negotiate unique tensions inherent in their position and may be required to develop novel understandings. To understand their contributions, our study investigates the nature of discourse within the Porn Free Women community. Drawing on a scripting approach to sexual behavior, we seek to identify the predominant themes of these discussions and to decipher the specific sexual scripts that these women collectively construct.

## Method

### Data Source

This study examined a dataset of submissions to the online discussion forum, Reddit. Reddit is a widely used platform with a global user base that comprises numerous forums or subreddits dedicated to areas of interest. Users within these subreddits can post submissions on particular topics and engage in hierarchically structured comments and responses. The Reddit community investigated in this study, Porn Free Women, is characterized by user anonymity, public accessibility for reading, and a substantial number of active users (13,000 members; r/pornfreewomen, 2023). Even so, when reporting research findings derived from this content, comments were decoupled from the author’s usernames which were replaced with arbitrary identifiers (e.g., S654) and unique phrases were paraphrased to ensure individual users could not be identified. Porn Free Women is not an isolated community; it is deeply embedded within the broader Reddit ecosystem, often influenced by the manosphere content prevalent on the platform. Notably, Porn Free Women is recommended on pornfree, a male-dominated subreddit, as a resource “for female porn addicts.” This cross-linking within Reddit highlights the interconnectedness of these communities. Porn Free Women is self-described as:

…a safe, women exclusive community for women of all walks of life working to overcome a PMO [pornography, masturbating, and orgasm] addiction or compulsion. Questions, experiences, opinions, struggles, and achievements are all welcome here. (r/pornfreewomen, 2023; [PMO definition added])

The data used for this study were sourced using the digital data aggregation service, Brandwatch. The subreddit was established on November 20, 2018, but submissions were only available for collection from January 2019 to February 9, 2023 (*N* = 8135). This collection of posts serves as the foundation for our analysis and comprises all available, public submissions to the forum. From these data, we explored the themes and scripts presented by Porn Free Women community members.

### Data Analysis

We used a mixed methods approach for data analysis to learn more about the sexual scripts discussed by women within the online anti-pornography movement. Natural language processing (NLP) through structural topic modeling (STM) was used to identify the prevalence of themes in the texts while a critical discourse analysis (CDA) of the submissions was used to examine the richer meanings present in the data.

#### Structural Topic Model

A computer-assisted NLP analysis was first conducted to identify the major themes present in the text of the submissions. Specifically, we fit a STM to the dataset. STM is a variant of the latent Dirichlet allocation (LDA) topic modeling algorithm, an unsupervised machine learning approach to finding associated elements within texts. These associations are then grouped by topics, which can help facilitate interpreting large sets of textual data (Roberts et al., 2019). Unlike the traditional LDA model, STM incorporates metadata associated with the text when identifying the frequency of topics (Roberts et al., 2019). For example, the type of submission (post vs. reply), Reddit score (the number of upvotes garnered by the post), and date of the submission were all included as structural covariates in the topic modeling algorithm. To effectively interpret topics, we used word association indices (e.g., probability, FREX, lift, and score), proportional statistics, and visualization tools for topic correlations provided in the *stm* package for R (Roberts et al., 2019). After STM topic identification, we analyzed the top 20 submissions related to each topic. Each author reviewed these comments and then collectively discussed levels of agreement on the salient themes per topic. After reaching a consensus on these themes, a second interpretation round was used to clarify and define the topic. Subsequently, according to definitions clarified in the previous stage and metadata provided by *stm*, the authors collectively correlated the topics, establishing a thematic hierarchy that organized the large document corpus into three distinct frameworks.

#### Critical Discourse Analysis

Our CDA is modeled from Beres (2014) integration of scripting theory with discourse analysis in exploring sexuality. This theory suggests that our understanding of sexuality, and the assumptions underpinning it, are reflected in societal discourses. For example, discourses around heterosexuality reveal foundational assumptions about its construction and representation within various cultural contexts. These assumptions emerge through distinct cultural scripts, which are not fixed but rather are dynamic and negotiable. This understanding of sexual scripts as fluid and subject to societal influences allows us to analyze the shaping of sexual behaviors and norms (Jackson & Scott, 2010). Recently, Aranda et al. (2021) offered a mixed methods approach, arguing that STM could supplement the traditionally thorough and targeted approach inherent in CDA. Informed by these pivotal studies, we advance the application of CDA in our research to dissect intersection of women’s discourse on pornography abstinence.

After identifying key themes about women’s perspectives in the Porn Free Women community through STM, we then focused on documents that specifically discuss sexual scripts. Within the three frameworks established by STM, we closely examined posts, leading to the identification of distinct sexual scripts under each framework. This approach facilitated the categorization of conceptual themes and the identification of distinct sexual scripts within three established frameworks. However, STM's limitations became apparent when it failed to reveal the intersections of these themes with broader systems and discourses. As such, these personal narratives were then singled out for further critical discourse analysis. Based on topics generated by the STM, we further explore “topic relations to derive broader meaning structures” and “linkages between discourses and context” (Aranda et al., 2021, p. 205). We meticulously reviewed all post-level (as opposed to replies) submissions (*n* = 843), selecting representative samples for in-depth analysis. This detailed examination of the data addressed STM’s limitations, particularly in capturing the interplay of various discourses. By analyzing the interaction of these sexual scripts and engaging in a deeper discourse analysis, we gained insights into the nuanced experiences of the members of the Porn Free Women community.

## Results

### Themes in Porn Free Women

**Fig. 1:**
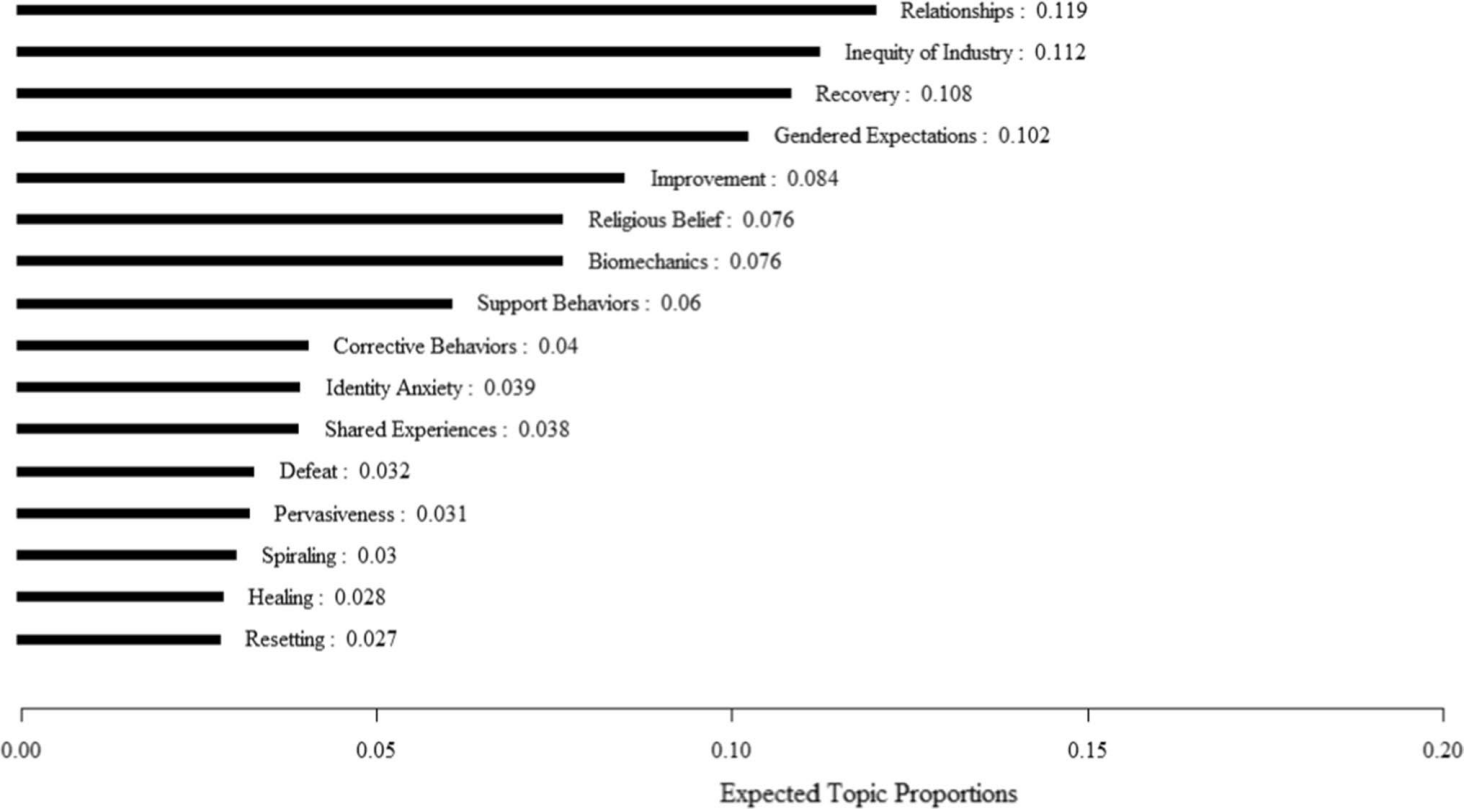
Expected structural topic modeling proportions in the porn free women forum

We generated 16 topics from the STM analysis. The results of the STM are presented in Fig. 1. The optimal number of topics was determined based on two diagnostic values: held-out likelihood and residual. To validate the categorized topics, we engaged in an iterative review of the STM output, ensuring a meaningful alignment of documents within respective topics. Our interpretations identified three overarching themes that fit these 16 topics. These themes offer a holistic understanding of how women discuss abstaining from pornography and address perceived addiction and problematic pornography consumption. By clustering granular topics into broader categories, we developed descriptive themes of the conversations in these communities (see Table 1).

**Table 1:** Structural topic modeling topics associated with overarching themes

Themes	Topics
Therapeutic	Recovery
	Improvement
	Religious belief
	Biomechanics
	Corrective behaviors
	Defeat
	Spiraling
	Healing
	Resetting Recovery
	Religious belief
Empowerment	Inequity of the pornography industry
	Support behaviors
	Pervasiveness of pornography
Heteronormative	Relationships
	Gendered expectations
	Identity anxiety

It is noteworthy that the STM did not identify these overarching themes; instead, the researchers bridged the granular topics to form a hierarchical framework of themes. The STM, likewise, did not identify attributes of the documents that permeated across topics. These common features included discussions about addiction to pornography, masturbation, and sex; the supportive nature within the community; and discussion of the purpose and regulation of the discussion forum such as restrictions on men posting to the forum. These discussions were present across the 16 topics; therefore, the three themes should be interpreted as contributing additional layers to the broader discussions of addiction, support, and community maintenance.

#### The Therapeutic Theme

The first theme, encompassing approximately half (50.1%) of the dataset, presented a therapeutic-oriented discussion. This theme positioned pornography abstinence as a journey of overcoming adversities, characterized by a process of healing and self-transformation. The submissions associated with this theme were underpinned by medical narratives which framed pornography as an addictive substance inflicting harm upon both the mind and body. Abstinence was seen as a way to remedy these damaging effects and “reset” unhealthy behaviors, echoing “rebooting” experiences identified by Fernandez et al. (2021). Spiritual or religious beliefs were occasionally incorporated into this discussion by constructing abstinence as a tool for personal growth or deepening one’s relationship with a higher power. Overall, this therapeutic theme, which combined narratives of medicalization, recovery, and religious belief, constituted the predominant perspective in the community.

#### The Heteronormative Theme

The second theme, representing 27% of the data, we interpreted as a heteronormative discussion. Recurring topics included advocacy for the “real” woman, mentions of romantic and familial relationships, and identity anxieties. A particular emphasis was placed on the role of pornography in heterosexual relationships, featuring dialogs about intimacy and relationship dynamics. These gendered expectations suggest an internalization of the heteronormative assumptions for appropriate ways of being a woman. Comments highlighted negotiations and expectations between authors and partners regarding intimacy, religion, and other aspects of relationships. Furthermore, debates over whether certain pornography consumption habits signify lesbian or gay sexualities were common, as were revelations of maintaining deceptive “double lives” by hiding pornography consumption from offline relationships. In sum, the pervasive influence of heteronormativity was evident in the construction and interpretation of abstinence. From this influence, identity and sexuality anxieties emerged when navigating desires that deviated from the dominant paradigm.

#### The Empowerment Theme

The third salient theme, accounting for 14.1% of the data, encapsulated a narrative of female empowerment within the context of pornographic culture. Participants shared personal encounters with sexually explicit content and the challenges of avoiding involuntary exposure to this content, which they described as psychologically “triggering.” In response, women sought to build a supportive community and shared practical strategies for personal well-being, including dietary supplements, medication, therapy, and using content-blocking tools. Within this theme, expressions of shared struggle and empathy were common, featuring mutual understanding and affirmations of the validity of feminist critiques of pornography. Particularly, participants connected pornography production and consumption with the objectification and exploitation of women. Overall, this theme highlights women’s agency in actively fostering communities that promote women’s health and morale.

### Sexual Scripts in Porn Free Women

The application of the STM in our analysis revealed specific, granular topics, but it did not inherently expose the overarching meta-themes that emerged. Additionally, the STM did not automatically recognize common document attributes that were prevalent across various topics and themes. Such attributes included self-reported instances of online sexual harassment and dialogs concerning the purpose and regulations of the discussion forum, particularly with regards to potential restrictions on posts made by men. Given the inherent constraints in STM to adequately accommodate the potential complexity of meaning embedded in texts, CDA serves as a valuable tool for enriching STM (Aranda et al., 2021). To balance, inform, and ground the datacentric and computational analysis offered by STM, we conducted further qualitative analysis that probed the intersections between the discussion themes. In our analysis, three distinct scripts were identified, namely the addiction script, heterosexual script, and liberation script. These scripts intertwined and intersected to form a multifaceted narrative surrounding women’s pornography abstinence.

#### Addiction Script

Our analysis elucidated the alignment of women’s narratives with the pervasive discourse on pornography addiction. As indicated by Burke and MillerMacPhee (2021), pornography addiction, undergirded by scientific validations drawn from neurobiology, has been often framed as a seemingly objective public menace. Men frequently deploy victimizing language to depict their adversity as sufferers of pornography addiction, thereby shaping a narrative of victimized masculinity (Chasioti & Binnie, 2021). Although women also use this victimizing rhetoric, they tend to identify themselves as victims of sexual violence and other forms of sexual trauma to rationalize their addiction. Furthermore, the addiction paradigm intensifies women’s anxiety and guilt, and simultaneously legitimizes stereotypes related to heterosexual behavior.

Firstly, the addiction script manifested as an embodiment of women’s suffering, reflecting the irrational ramifications of sexual trauma. Pornography addiction was often portrayed as a deleterious consequence of women’s sexual experiences within pornographic culture. For some women, pornography may serve as an emotional salve to mitigate or numb the distress of past traumas. For instance, one woman (S564) recounted her childhood experiences of being mistreated by her mother, and how her addiction to pornography served a way to vent her anger and frustration from years of mistreatment. Similarly, another woman traced the roots of her struggle with pornographic fantasy to her childhood sexual abuse. Furthermore, many women identified themselves as victims of their partners’ pornography addiction. For instance, one woman (S345) narrated her painful experiences of fulfilling her partner’s addictive needs, critiquing that “porn addiction is real and it leads people to commit horrible, selfish acts toward their partners because they stop seeing them as human beings.” Here, the validation of the concept of pornography addiction was attributed more to their relational suffering rather than their personal experiences.

Upon self-identifying as pornography addicts, women become increasingly vulnerable to a coexisting framework of self-regulation and social norms. The phenomenon of self-diagnosis of addiction has surged in recent years, particularly within the neoliberal paradigm that stipulates individual conduct (Zou et al., 2023). Within the prevailing therapeutic milieu, the label of an “addict” carried considerable implications, suggesting an individual’s surrender to excessive desires. A case in point, a woman (S67), self-identified as an addict, asserted, “I will conquer and quell this passion; the passion shall not rule over me!” Her words framed addiction as an enemy to be battled and defeated, reflecting the existence of a dominant moral code that regarded uncontrolled desire as deviant. This internal battle is often intensified by the language women use to describe themselves. Statements like “I should’ve controlled myself” and “I need to feel like I’m not a monster” (S98) reveal a harsh self-criticism, influenced by societal norms that label uncontrolled desire as deviant. Furthermore, the label of “addicts” frequently co-occurred with derogatory labels such as “slut” or “cheap women” in their narratives, insinuating blame and reflecting societal pressures to regulate female sexual behavior.

Consequently, women often reported guilt and anxiety when discussing their struggles with pornography addiction, even with close families and friends. For example, one participant (S32) detailed her dwindling self-esteem tied to her intense sexual drive and struggles with pornography addiction, asserting, “I’ve been struggling badly with my mental health recently, and an aspect of it that keeps coming up is self-loathing over the pornography I used to watch, even though I’m mostly clean now.” This scene aligned with traditional sexual scripts that presupposed women as sexually passive, favoring relational sex, thereby fostering guilt and shame internalization among female pornography consumers.

However, it is noteworthy that some women expressed empowerment through overcoming their addiction and expressed an aspiration to acknowledge and validate its existence as a crucial aspect of their identity. A participant (S49), for example, recognized her enduring addiction, expressing a desire to embrace its existence as an essential aspect of her identity, stating, “The addiction is still alive in me and it always will be…I’m learning to make peace with that. It’s a part of me and deserves to be given space, to be accepted and loved and nourished.” This viewpoint challenged the dominant moral and social norms which generally stigmatize addiction and assign blame to the addict. Rather, it signified a shift from viewing addiction as a personal failure to understanding it as a part of the individual’s identity that demands acceptance.

#### Heterosexual Script

The impact of heteronormativity and its role in shaping heterosexual subjectivity among women emerged as a significant element of these narratives. The women expressed an internalized societal expectation and norm surrounding heterosexuality, underscoring the interactional nature of heterosexual relationships (Sun et al., 2016). Consequently, these women prioritized their relationships and experienced identity distress when their pornography fantasies diverged from the heterosexual script.

Many women reported guilt associated with pornography consumption, especially when they engaged in romantic relationships. Influenced by heterosexual scripts, women were expected to regulate their sexuality and sexual encounters. For example, one woman (S657) wrote, “My partner knows I have had issues with pornography and doesn’t mind, but it’s so wrong and I feel like I am betraying my partner.” Her choice of words (“betraying”) suggested disloyalty and dishonesty, indicating her internalization of the notion that pornography consumption was a transgression incompatible with fulfilling the role of a committed partner. The societal pressure to embody the “morally superior” female partner is also evident, as in the case of participant (S321), who voiced shame about struggling with what she identified as “a man’s issue.” This phraseology insinuated that societal expectations precluded women from confronting sexual desires or behaviors and imposed a gendered standard for sexual morality.

Some women reported using pornography to fulfill their need for intimacy and relationship, aligning with the societal expectations propagated by heterosexual scripts that emphasize the importance of romantic relationships. For instance, one woman shared her struggle with pornography addiction writing, “I don’t want porn, I want someone whom I care about to have an intimate bond with emotionally and physically.” Here, she perceived the attention garnered from pornography as a pseudo facsimile of authentic romantic engagement, reflecting the broader patterns of socialization for women to highly value romantic relationships. In contrast, it’s been observed that heterosexual men may champion abstention from pornography to underscore real sexual experiences as badges of masculinity (Taylor & Jackson, 2018). However, within women’s narratives, the motivation for recovery from pornography addiction primarily hinges on the expectation of fulfilling romantic relationships, as opposed to sexual experiences.

Moreover, the decision to refrain from pornography among women was often motivated by identity anxieties arising from deviations against heteronormative expectations. Specifically, women who exhibited a preference for lesbian pornography despite identifying as heterosexual experienced considerable distress. As demonstrated by one member, “I could only orgasm to lesbian porn though I was attracted only to men in real life. I don’t like the idea of being with a woman.” This incongruity between her desires and self-identification creates anxiety surrounding her self-perception and sexuality. Similarly, individuals identifying as queer grappled with internal conflicts arising from the dissonance between their private erotic interests and the inflexible delineations of sexual identity categories. For example, a woman wrote, “I have consumed other types of pornography, which was not just lesbian (bi, trans, hetero, gay). Now, I’m wondering if that makes me stop being a lesbian…” Despite her wide-ranging attractions, she questioned whether her diverse consumption invalidated her self-identification as a lesbian. Her concern underscored the societal pressure to conform to rigid sexual identity labels, neglecting the potential fluidity and multiplicity of individual desires or behaviors. Taken together, these narratives illuminated the potential conflict and distress elicited when personal experiences failed to conform to the heteronormative dictates of the prevailing societal structure.

#### Liberation Script

Despite the entrenched misogyny within pornography abstinence forums (Taylor & Jackson, 2018), there has been a notable upsurge in feminist discourse within female-centric anti-pornography communities. These communities emerge as sanctuaries for women to discuss and share their experiences with pornography, highlighting the necessity for support structures that counter the prevalent misogyny and online harassment faced by women. Furthermore, the narratives shared in the forum illuminated the complex interplay between feminist ideology and the pornographic content consumption.

The increasing prevalence of posts advocating for “women-only communities” reflected an escalating interest in establishing secure and supportive environments where women connect, share resources, and empower each other. A prevailing atmosphere of misogyny and online harassment often deters women from engaging in certain online communities, particularly those associated with pornography. Many women reported revulsion toward social media platforms and their respective algorithms. For instance, one woman (S78) criticized social media for its pervasive misogyny, stating: “as an addict, it was so triggering. I couldn’t go on Reddit and type in a single letter without 10 porn subs coming up. And degrading, atrocious, objectifying shit from pathetic men who don’t value women.” Many women members shared their experiences of virtual harassment and violence, with one (S213) saying, “I couldn’t even express my troubles without some weirdo making me feel uncomfortable.” The evolution of harassment in the digital landscape, from verbal abuse to non-verbal forms including images, videos, and symbols, was cited by members as a factor contributing to their discomfort. Male supremacists’ justification of networked harassment based on alleged victimization of feminists further exacerbates this issue (Han & Yin, 2023). Women-only anti-pornography communities thus emerged as havens where women can safely negotiate their erotic subjectivities.

Many participants expressed narratives aligning with anti-pornography feminist ideology, contending that pornography fostered the exploitation and oppression of women. (Bridges et al., 2010). One woman (S292) connected her decision to quit pornography with radical feminist ideas:

Another huge influence to give up porn comes from the ideas of radical feminism—that you cannot know where your porn comes from, whether or not it’s done by consenting adults, and that so much of porn is violent towards women and drowns men (especially younger boys) in unrealistic, potentially dangerous bullshit. (S292)

Empirical evidence corroborated the frequent portrayal of violence against women in mainstream pornography (Bridges et al., 2010). Participants voiced their disgust toward such aggressive pornography and disclaimed the “exploitative nature” (S349) of the pornography industry and the “sexual degradation and humiliation” (S28) depicted in pornographic videos. Similarly, some women disapproved of the perpetuation of harmful stereotypes in pornography. For example, one woman developed new anxieties about her body after comparing herself to the beauty standards portrayed in pornography. She argued, “Now I can’t stand to look at porn anymore it is such a misinterpretation of what sex should mean to us a society or as women. I loathe the porn industry.” In these instances, women positioned themselves as active agents rejecting the objectification of women’s bodies for the satisfaction of the male gaze.

Interestingly, some women experienced guilt when consuming pornography, more because of the industry’s mistreatment of women rather than the previously mentioned heterosexual scripts. As one woman (S457) stated:

Like I really liked BDSM, rough and occasionally hardcore porn and even though I don’t use Pornhub or anything anymore I’m just struggling so much with guilt and self-hatred. I feel like a horrible abusive person and I’m so scared of the possibility that the actresses in these videos may not have been acting/ been forced or manipulated/ been underage without me knowing […] I feel like a fraud who acts like a feminist and progressive person but is actually just evil. (S457)

Several other narratives corroborated this sentiment, indicating preferences for BDSM but expressing guilt catalyzed by their interpretation of feminist ideas. In these scenarios, women’s pornography consumption seems not only impeded by societal norms but also curtailed by their understanding of feminist doctrines that decry harmful pornographic material. The use of strong emotive language (“loathe,” “struggling so much with guilt and self-hatred,” “horrible abusive person”) reflects an intense internal conflict and a deep concern for the ethical implications of their actions. This struggle is often magnified by personal experiences with sexual violence, leading to a heightened sensitivity to coercion and exploitation in pornographic content (Parvez, 2006). Women in these communities are not only navigating societal expectations but also grappling with the implications of their consumption choices on their feminist ideals.

## Discussion

In this study, we shine light on women’s narratives regarding pornography abstinence. We engage in an exploratory investigation of Reddit posts from the Porn Free Women forum, utilizing a mixed methods approach to delineate the themes and sexual scripts presented in these discussions. Our analysis unveils three key themes: the therapeutic theme that interprets pornography abstinence as a means of addressing unhealthy behaviors; the women’s empowerment theme that places pornography abstinence as part of a journey toward autonomy; and the heteronormative theme that positions pornography abstinence as means of preserving femininity. A qualitative analysis of representative posts also illuminates the coherence between the sexual scripts discussed and traditional sexual scripts outlined in prior literature.

When compared to prior research, our findings identify both similarities and striking differences in women’s discourses on pornography abstinence compared to men’s narratives. Aligning with Zimmer and Imhoff’s (2020) findings, which suggest men’s motivation for abstinence is predominantly rooted in the perception that masturbation adversely affected their health, our research similarly demonstrates that women rationalize abstinence by referring to the addictive potential of pornography. This addiction narrative interacts intricately with the heterosexual script, thereby amplifying women’s experiences of anxiety and guilt associated with pornography consumption. Moreover, the victimization rhetoric adopted by women often manifests in self-portrayals as victims of their past trauma and partner-inflicted sexual abuse. Contrastingly, men often depicts themselves as victims of extrinsic factors, such as perceived feminist and capitalist conspiracies (Burnett, 2022; Zou et al., 2023).

Furthermore, earlier research suggests that men’s motivation for abstinence was largely associated with their desire to engage in real sex to display their masculinity (Taylor & Jackson, 2018). However, our findings suggest that women who engaged in pornography abstinence would place greater emphasis on fostering traditional romantic relationships over pursuing sexual encounters. Thus, our research suggests women internalized heterosexual scripts which heightened emphasis on relationships. Given the prevailing influence of heterosexual scripts, the findings of our study are not entirely surprising. Echoing Spišák’s (2017) qualitative analysis, we likewise discern that pornography consumption that deviates from heteronormativity emerged as a source of identity-related anxiety, which could potentially motivate pornography abstinence among heterosexual women. Our findings not only illuminate the experiences of heterosexual women but also highlight the need to extend this analysis to genderqueer individuals. Notably, our study suggest that queer individuals may experience inner anxieties due to the monotonous standard of pornography consumption entrenched in society. This highlights the pressing need for broader and more inclusive dialogs on sexuality that empower individuals to embrace their authentic identities.

Earlier research has emphasized the mediating role of religiosity and moral values in constructions of pornography addiction (Grubbs et al., 2017). Studies focused on heterosexual men’s narratives propose that religious discourses provide a valuable framework that facilitates the preservation of personal integrity and autonomy in contexts of felt powerlessness (Chasioti & Binnie, 2021). Despite religious references appearing in only 7.6% of our data, it is notable that such beliefs also frequently coincided with notions of addiction. This observation aligns with the findings of Chasioti and Binnie (2021), underscoring the significant interplay between religion and perceptions of addiction. One notable distinction observed was the propensity of women to invoke a higher power, often religion, as a rationalization for their motivation to overcome addiction. This variance indicates that different gendered experiences may shape the interaction of religion and perceptions of addiction.

Our research responds to recent calls to examine adherence to and transformation within heterosexual sexual scripts (Klein et al., 2019). Particularly, we delve into the intricate manner in which addiction scripts and liberation scripts interact with and intersect the conventional heterosexual script, consequently forming a complex narrative around women’s abstinence. In particular, we observe a notable prevalence of liberation scripts. Our findings indicate that women are actively resisting the heterosexual scripts propagated by pornographic material, a medium notorious for reproducing and reinforcing gender stereotypes. We also note the multifaceted role of addiction scripts within this context. On one level, they provide a pathway toward acceptance, facilitating empowerment and autonomy for women amidst conventional sexual norms. Simultaneously, when juxtaposed with the heterosexual script, these addiction scripts offer a regulatory framework that governs female sexual behavior. Importantly, our analysis introduces addiction and liberation scripts as newly identified elements within this dataset. However, their interaction with traditional heterosexual scripts is far from disjointed. Rather, these scripts collectively contribute to the narrative landscape surrounding women’s experiences in the pornography abstinence movement.

Women’s engagement with pornography is characterized by a notable ambivalence. While it serves as a source of entertainment, arousal, and education for some (Nikunen, 2007), this enjoyment often comes with internal conflict and discomfort. This conflict is a form of “emotional labor,” as described by Parvez, (2006), marked by intense reflectivity and emotional struggle during consumption. Parvez (2006) attributes women’s discomfort partly to concerns about the well-being and authenticity of porn actresses’ pleasure and their personal experiences with sexual violence. Expanding on this, our research reveals that women’s emotional labor in consuming pornography is also influenced by their feminist beliefs. This aligns with Ciclitira’s (2004) observation of the feminist “sex wars,” which profoundly impacted views on pornography and feminism. Ciclitira highlights that historical accounts from the 1970s and 1980s reflect a struggle within women, torn between their feminist beliefs and enjoyment of pornography. In response to the polarized debates of the past, which Ciclitira notes have led to some women’s feelings of alienation, some scholars propose moving beyond binary frameworks like disgust versus pleasure and pro- versus anti-pornography (Gurevich et al., 2017; Paasonen, 2007). Our analysis also supports moving beyond this binary framework. Even for women dedicated to pornography abstinence, their collectively constructed scripts make sense of their decision through intersecting and sometimes competing narratives. Standing on the social constructionist position, our research showed how heterosexual norms, feminist perspective, and medical frameworks epitomize women’s view and sense-making on pornography abstinence.

In the Porn Free Women forum, women’s empowerment manifests in two key ways. Firstly, “empowerment as choice” is significant, emphasizing the development of self-efficacy and the belief in one’s ability to achieve goals, which parallels prior research on other online women’s pornography abstinence forums (Ammari et al., 2022). This self-belief is crucial in overcoming addiction and often leads women to form new identities through recovery. Secondly, empowerment emerges through community support, also paralleling prior research (Ammari et al., 2022), where women share experiences and support each other, creating a collective network of strength and resilience.

However, this articulated empowerment must be examined in broader sociocultural and toxic sociotechnical environments. As Gavey (2012) and Foucault’s (1994) concept of “panoptic modality of power” (p. 211) suggest, true empowerment in overcoming addiction considers broader societal structures. Women’s struggles with pornography are often intertwined with sexual desires, relationships, and life experiences. While overcoming addiction is perceived as a process of self-discovery and strengthening self-efficacy, it is crucial to acknowledge the impact of societal structures like neoliberal and heterosexual discourses on women’s conflicting feelings. Moreover, empowerment through community is complicated by the fact that most pornography abstinence forums are male-dominated and often misogynistic. Even as women carve out new spaces for support, they face online harassment, a situation worsened by the design of platforms like Reddit, which inadequately address objectionable content (Massanari, 2017). This situation underscores the need for future research to explore how women’s empowerment in online spaces is shaped and constrained by the interplay of broader sociocultural frameworks and the governance policies of platforms in patriarchal societies.

### Implications

This study has several noteworthy implications. Firstly, it enhances our comprehension of pornography abstinence experiences by incorporating women’s perspectives, which have often been overshadowed by a predominant focus on men’s discourse in online communities in prior research. Secondly, our research employs a mixed methods approach combining computation-aided analysis and critical discourse analysis, illuminating the phenomenon of pornography abstinence and expanding the conceptual and analytical scope of discourses surrounding online anti-pornography movements. Although Osadchiy et al. (2020) conducted a large-scale analysis of over 20,000 Reddit posts from the NoFap forum, we build on this work by adding a comprehensive qualitative understanding of the Porn Free Women community. Moreover, our study presents an analysis of sexual scripts in the context of women’s perceptions of pornography as risky and thereby opting for abstinence. This provides an alternative point to trends in literature that largely explore women’s consumption of pornography from an empowerment perspective. Furthermore, our findings begin to articulate the intersectional nature of the sexual scripts presented on Porn Free Women, and we call for continued work to diversify the discourse on pornography and sexuality. This push for inclusivity acknowledges and validates the range of experiences, priorities, and identities within the realm of sexual expression.

### Limitations and Directions for Future Research

Although the current study consists of a large, real-world sample, it is not representative of the global population. As our data are collected from a single website, our sample is biased in ways consistent with the forum’s user base (i.e., English-speaking Reddit users). In this regard, the skew toward women within our sample could merely be a product of the demographic composition of Reddit.

Further insights could benefit from broader dataset that includes men’s perspectives and cross-cultural considerations. Specifically, comparing pornography abstinence between women-dominated and men-dominated communities through integrating STM and CDA analyses could further illuminate differences. Similarly, an exploration of alternative sexual scripts across different cultures can provide insights into how cultural constructions shape attitudes toward pornography, challenging universalist claims. Further research is also needed to distinguish the nuanced differences between the pornfree and NoFap communities, which each have forums that express ideals of gender inclusivity while the discussion is practically dominated by men. Each community also features forums created as spaces for women as reactions to the gendered imbalance of the discussions. Longitudinal studies would be particularly beneficial in understanding how concepts of sexuality and relationships evolve within these online spaces, especially in the context of the ever-changing social and technological landscape. It is crucial to investigate and theorize how sexual scripts are communicated, negotiated, and enacted within the dynamic context of the digital era, characterized by a plethora of social and dating applications.

### Conclusions

Although traditionally dominated by men and masculine perspectives, the online pornography abstinence movement has attracted a substantial number of women. These women negotiate unique tensions and, through their discussions, articulate novel expressions of sexual scripts. However, current literature largely ignores women’s perspectives in these movements. Through structural topic modeling and discourse analysis, we find that the women who join this movement and discuss their experiences on Porn Free Women negotiate their perspectives in ways distinct from men in these movements and the broader manosphere of online discussion reported by prior research. By integrating our findings with the theoretical framework of sexual script theory, we further illustrate the complex intersections of addiction, heteronormativity, and liberation scripts. This intersectionality unveils the resurgence of deep-seated societal tensions within the digital media landscape and online discussions. Our research thus contributes to a richer, more nuanced understanding of the dynamics at play within the pornography abstinence movement, and underscores the necessity of a more gender-inclusive discourse.

In closing, we stress the importance of attending to narratives that women share about their preferences and experiences, whether they pertain to engaging with pornography or choosing pornography abstinence. These narratives, which are often marginalized or trivialized in online spaces, hold substantial value for a more comprehensive understanding of women’s sexual experiences. Further, our research underscores the issue of women’s marginalization in the online manosphere. Through our investigation, we identify concerted efforts by women to carve out safe spaces within the manosphere, often marked by pervasive misogynistic narratives. These discussions can be seen as strategic attempts to construct empowering narratives of womanhood that actively challenge the prevailing masculinity-oriented discourse. However, we find that these attempts may be constrained by the online harassment endemic in these spaces, which is reported to silence individuals and inhibiting them from expressing their opinions (Nadim & Fladmoe, 2021). Thus, the outcomes of our study amplify the call for critical interventions and protective measures to facilitate the expression of diverse voices within such digital environments, ultimately fostering a more inclusive and respectful online discourse.

## Data Availability

Anonymized data available from the authors by request.

## Code Availability

Available from the authors by request.
